# Diabetes treatments and risk of heart failure, cardiovascular disease, and all cause mortality: cohort study in primary care

**DOI:** 10.1136/bmj.i3477

**Published:** 2016-07-13

**Authors:** Julia Hippisley-Cox, Carol Coupland

**Affiliations:** Division of Primary Care, University Park, University of Nottingham, Nottingham NG2 7RD, UK

## Abstract

**Objective** To assess associations between risks of cardiovascular disease, heart failure, and all cause mortality and different diabetes drugs in people with type 2 diabetes, particularly newer agents, including gliptins and thiazolidinediones (glitazones).

**Design** Open cohort study.

**Setting** 1243 general practices contributing data to the QResearch database in England.

**Participants** 469 688 people with type 2 diabetes aged 25-84 years between 1 April 2007 and 31 January 2015.

**Exposures** Diabetes drugs (glitazones, gliptins, metformin, sulphonylureas, insulin, other) alone and in combination.

**Main outcome measure** First recorded diagnoses of cardiovascular disease, heart failure, and all cause mortality recorded on the patients’ primary care, mortality, or hospital record. Cox proportional hazards models were used to estimate hazard ratios for diabetes treatments, adjusting for potential confounders.

**Results** During follow-up, 21 308 patients (4.5%) received prescriptions for glitazones and 32 533 (6.9%) received prescriptions for gliptins. Compared with non-use, gliptins were significantly associated with an 18% decreased risk of all cause mortality, a 14% decreased risk of heart failure, and no significant change in risk of cardiovascular disease; corresponding values for glitazones were significantly decreased risks of 23% for all cause mortality, 26% for heart failure, and 25% for cardiovascular disease. Compared with no current treatment, there were no significant associations between monotherapy with gliptins and risk of any complications. Dual treatment with gliptins and metformin was associated with a decreased risk of all three outcomes (reductions of 38% for heart failure, 33% for cardiovascular disease, and 48% for all cause mortality). Triple treatment with metformin, sulphonylureas, and gliptins was associated with a decreased risk of all three outcomes (reductions of 40% for heart failure, 30% for cardiovascular disease, and 51% for all cause mortality). Compared with no current treatment, monotherapy with glitazone was associated with a 50% decreased risk of heart failure, and dual treatment with glitazones and metformin was associated with a decreased risk of all three outcomes (reductions of 50% for heart failure, 54% for cardiovascular disease, and 45% for all cause mortality); dual treatment with glitazones and sulphonylureas was associated with risk reductions of 35% for heart failure and 25% for cardiovascular disease; triple treatment with metformin, sulphonylureas, and glitazones was associated with decreased risks of all three outcomes (reductions of 46% for heart failure, 41% for cardiovascular disease, and 56% for all cause mortality).

**Conclusions** There are clinically important differences in risk of cardiovascular disease, heart failure, and all cause mortality between different diabetes drugs alone and in combination. Overall, use of gliptins or glitazones was associated with decreased risks of heart failure, cardiovascular disease, and all cause mortality compared with non-use of these drugs. These results, which do not account for levels of adherence or dosage information and which are subject to confounding by indication, might have implications for prescribing of diabetes drugs.

## Introduction

Cardiovascular disease and heart failure are major causes of morbidity and mortality in people with type 2 diabetes.^1^ ^2^ Once heart failure is present in people with diabetes, mortality is increased 10-fold and five year survival is only 12.5%, a prognosis worse than for metastatic breast cancer.^2^ Several diabetes drugs have been associated with an unexpected increase in risk of heart failure during both clinical trials^3^ and post-marketing surveillance raising concerns about the overall risks and benefits for people with diabetes.^4^ ^5^

Following its launch in 2000,^6^ rosiglitazone, the first drug in the “insulin sensitising” thiazolidinedione class, was associated with an increased rate of heart failure.^5^ This resulted in its withdrawal from Europe, India, New Zealand, and South Africa in 2010-11. Rosiglitazone is, however, still prescribed in the United States—a controversial decision^7^ informed by the open label “non-inferiority” rosiglitazone evaluated for cardiovascular outcomes in oral agent combination therapy for type 2 diabetes (RECORD) trial funded by the manufacturers of rosiglitazone (Avandia; GlaxoSmithKline).^4^ The RECORD study assessed cardiovascular outcomes in 2220 people prescribed rosiglitazone in combination with either metformin or sulphonylureas compared with 2227 prescribed both metformin and sulphonylureas between 2001 and 2008. Although the numbers of events were low, the trial reported an increased risk of heart failure with rosiglitazone and was unable to rule out an increased risk of myocardial infarction.^4^ The design, results, and interpretation of this trial have been heavily criticised.^8^

Pioglitazone is another thiazolidinedione that decreases blood glucose levels. The placebo controlled PROactive trial of pioglitazone^9^ was also controversial in its design and interpretation, largely because of the choice of composite endpoints.^10^ Although the trial failed to clearly show improved cardiovascular outcomes for patients, it reported increased hospital admissions for heart failure as an adverse effect,^9^ as did a subsequent meta-analysis of 8554 patients prescribed pioglitazone.^11^ Meanwhile, a Canadian cohort study comparing pioglitazone with rosiglitazone between 2002 and 2008 reported a lower risk of heart failure and death in pioglitazone users.^12^ Similarly, a US cohort study reported a lower risk of stroke, heart failure, and all cause mortality among patients prescribed pioglitazone compared with rosiglitazone.^13^ Pioglitazone continues to be prescribed in the United Kingdom and the US although it has been withdrawn elsewhere owing to concerns about an increased risk of bladder cancer.^14^

Dipeptidyl peptidase-4 (DPP-4) inhibitors, also known as gliptins, are a relatively new class of diabetes drug that are included in international guidelines^15^ as second line agents after metformin, although data on long term clinical benefits and safety are inconclusive.^16^ In a placebo controlled clinical trial, saxagliptin was associated with an unexpected 27% increase in admissions to hospital from heart failure.^3^ In the Examination of Cardiovascular Outcomes with Alogliptin versus Standard of Care (EXAMINE) trial ,^17^ alogliptin did not significantly increase overall risk of hospital admissions for heart failure compared with placebo. Similarly, in the Trial Evaluating Cardiovascular Outcomes with Sitagliptin (TECOS) trial, sitagliptin was not associated with an increased risk of heart failure compared with placebo.^18^ Observational studies have also found inconsistent associations. Sitagliptin was associated with an increased risk of heart failure in a cohort study of 8288 Taiwanese patients using sitagliptin over 1.5 years.^19^ Conversely, a US cohort study of 8032 sitagliptin users showed no excess risk of hospital admission or death compared with users of other glucose lowering agents.^20^ Although heart failure was included within the composite endpoint, it was not evaluated separately. A meta-analysis of 25 trials of 7726 patients receiving sitagliptin or a comparator agent between 12 weeks and two years was undertaken by the manufacturers of sitagliptin (Januvia; MSD).^21^ The composite study endpoints included major adverse cardiovascular events (defined as ischaemic events or cardiovascular deaths), but the study did not specifically evaluate risk of heart failure. In summary, the findings from clinical trials and observational studies are inconsistent, which may reflect differences in study design, study duration, individual drugs, or outcome measures.

Uncertainty remains over the longer term comparative risks among patients prescribed different diabetes drugs, particularly gliptins and glitazones alone and in combination with other diabetes drugs.^22^ ^23^ Regulatory agencies have responded to this uncertainty by requiring evidence that new diabetes drugs are not associated with harmful increases in cardiovascular events rather than the more stringent requirement that the drugs result in evidence of clinical benefit.^24^ ^25^

Concerns have also been raised about the safety of sulphonylureas, an older class of oral diabetes drug, as these have been linked with increased adverse cardiovascular events in some^5^ but not all studies.^26^ The lifelong nature of diabetes, the noticeable increase in its incidence and prevalence, and prescribing recommendations in guidelines,^15^ mean that the number of people prescribed diabetes drugs is likely to increase. Given the impracticability and ethical difficulties of head-to-head trials comparing different agents, the risks of clinical outcomes need to be quantified in large representative populations of people prescribed these drugs over longer periods. This information, which is available from large longitudinal observational databases, can complement information from meta-analyses of clinical trials that, while valuable, are prone to publication bias and lack sufficient detail, duration of follow-up, or the power to make relevant comparisons for unintended effects.^21^ ^23^

We therefore carried out a cohort study using a large UK primary care database with linked general practitioner, mortality, and hospital admissions data to investigate the associations between different classes of diabetes drugs and the risks of heart failure, cardiovascular disease, and all cause mortality for people with type 2 diabetes. We were particularly interested in the risks associated with the newer agents, including glitazones and gliptins. In a companion paper we reported on a similar analysis examining the risks of microvascular complications (severe kidney disease, blindness, lower limb amputation), hyperglycaemia, and hypoglycaemia between different classes of diabetes drugs in people with type 2 diabetes.^27^

**Figure 1:**
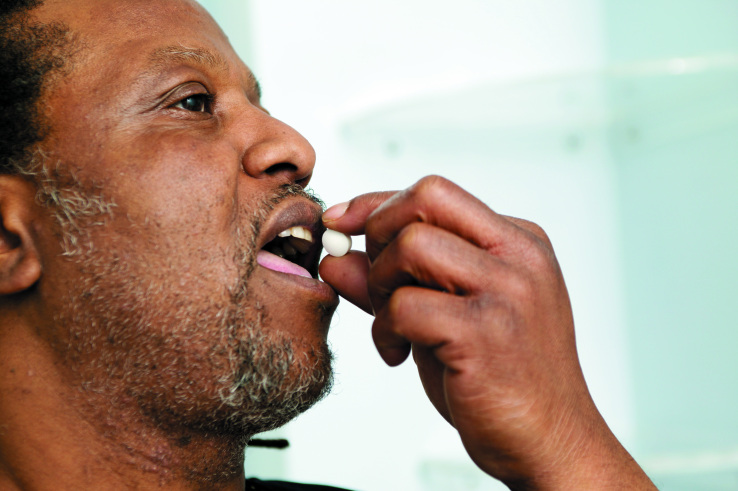


## Methods

We did a population based open cohort study of people in England aged 25-84 years with a diagnosis of type 2 diabetes. We used a large population of primary care patients derived from version 40 of the QResearch database (www.qresearch.org). QResearch is a continually updated patient level pseudonymised database with event level data extending back to 1989. QResearch currently includes clinical and demographic data from over 1243 general practices in England and two practices in Scotland, covering a population of over 24 million patients, and collected in the course of routine healthcare by general practitioners and associated staff. The primary care data include demographic information, diagnoses, prescriptions, referrals, laboratory test results, and clinical values. Diagnoses are recorded using the Read code classification.^28^ QResearch has been used for a wide range of clinical research, including the assessment of unintended effects of commonly prescribed medicines.^29^ ^30^ ^31^ ^32^ ^33^ ^34^ The primary care data are linked at individual patient level to hospital episode statistics and mortality records from the Office for National Statistics. Hospital episode statistics provides details of all National Health Service inpatient admissions since 1997, including primary and secondary causes coded using the ICD-10 (international classification of diseases, 10th revision) classifications and OPCS-4 (Office of Population Censuses and Surveys, fourth revision) codes for operations and interventions. ONS provides details of all deaths in England with primary and underlying causes, also coded using the ICD-10 classification. Patient records are linked using a project specific pseudonymised NHS number, which is valid and complete for 99.8% of primary care patients, 99.9% of ONS mortality records, and 98% of hospital admissions records.^1^

### Inclusion and exclusion criteria

The study included all QResearch practices in England that had been using the Egton Medical Information Systems (EMIS) computer system for at least a year. We initially identified an open cohort of people aged 25-84 years registered with eligible practices between 1 April 2007 and 31 January 2015. This time interval was chosen because both pioglitazone and gliptins were available in the UK during the full study period. We then selected people with diabetes if they had a Read code for diabetes or more than one prescription for a diabetes drug.

We excluded people as having type 1 diabetes if they had received a diagnosis aged less than 35 and had been prescribed insulin.^35^ We also excluded patients without a postcode related deprivation score. For each patient we determined an entry date to the cohort, which was the latest of the following: date of diagnosis of diabetes, 25th birthday, date of registration with the practice plus one year, date on which the practice computer system was installed plus one year, and the beginning of the study period. To reduce bias we used an incident user design for people prescribed glitazones, gliptins (our main drugs of interest), or insulin.^36^ As in other studies,^20^ we defined incident users as people without a prescription for these drugs in the 12 months before the study entry date, and we excluded people who had received any of these drugs in the previous 12 months. We included prevalent users of metformin or sulphonylureas in the study cohort; if we had excluded them the numbers of new users of glitazones and gliptins—our main exposures of interest—would have been substantially reduced because these drugs are usually prescribed after monotherapy with metformin or sulphonylureas. People with an existing diagnosis of an outcome of interest at the study entry date were also excluded from the analysis of that outcome. Patients were censored at the earliest date of the first recorded diagnosis of the outcome of interest, death, deregistration with the practice, last upload of computerised data, or the end date of the study (31 January 2015).

### Outcomes

Our primary outcomes were incident heart failure, cardiovascular disease, and all cause mortality, recorded in either the patient’s primary care record, linked hospital record, or mortality record.

#### Definition of outcomes

We used Read codes to identify recorded diagnoses of heart failure from the primary care records (G58%, G5yy9, G5yyA, 662f, 662g, 662h, and 662i). To identify incident cases of heart failure from hospital and mortality records, we used ICD-10 clinical codes (I110, I130, I42, and I50). We used the earliest recorded date of heart failure on any of the three data sources as the index date for the diagnosis of heart failure.

Our definition of cardiovascular disease included coronary heart disease (angina and myocardial infarction), stroke, or transient ischaemic attacks but not peripheral vascular disease. The supplementary file lists the Read codes used for case identification on the primary care record. The ICD-10 codes used for case identification on the ONS death certificate or hospital admission records were: angina pectoris (I20), acute myocardial infarction (I22), complications after acute myocardial infarction (I23), other acute ischaemic heart disease (I24), chronic ischaemic heart disease (I25), and ischaemic stroke (I63, I64) or transient ischaemic attack (G45). We used the earliest recorded date of cardiovascular disease on any of the three data sources as the index date for the diagnosis of cardiovascular disease.

All cause mortality was defined by the status of death recorded in the general practice systems linked to the date and cause of death as recorded on the ONS mortality record.

### Exposure data

Our primary exposures of interest were new use of gliptins and new use of glitazones during the study period. For each participant we extracted details of all individual prescriptions for all types of diabetes drug, including the prescription date and the type of drug. We partitioned the follow-up time into treatment periods, where each period corresponded to treatment with a particular type or combination of diabetes drug, or could be a period of no treatment with any diabetes drugs. If the patient changed to a different type of treatment or to a different combination of treatments, we classified that as a separate treatment period. For example, if a patient was prescribed metformin alone on entry to the cohort for 12 months and then was prescribed both glitazones and metformin for a further 24 months and then had a treatment free period for six months until they were censored, they would have three treatment periods (metformin only for 12 months, metformin and glitazones for 24 months, and no treatment for six months).

We determined the duration of each treatment period by calculating the number of days between the earliest issue date and the latest issue date plus 90 days for the type of treatment prescribed. If another treatment was added before the initial treatment was stopped then we calculated the duration of the treatment period on the initial treatment alone to be the number of days between the earliest issue date for the initial treatment and the earliest issue date for the next treatment. We added 90 days to the last prescription date as an estimate of the date on which the patient stopped treatment (the “stop date”), we made this assumption to allow for events that occur during a withdrawal period to be attributed to the drug rather than being counted as unexposed time. For the analysis, we used six binary exposure variables for each treatment period to indicate treatment with any of the diabetes drugs, grouped into six drug classes—glitazones (including rosiglitazone and pioglitazone), gliptins, metformin, sulphonylureas, insulin, and other oral diabetes drugs (including α-glucosidase inhibitors, sodium-glucose cotransporter 2 inhibitors, glinides, guar). This accounted for patients receiving different combinations of these drugs during a treatment period. To further assess associations for different specific treatment combinations (such as dual treatment with metformin and glitazones) we also categorised treatments during each treatment period into one categorical variable with 21 mutually exclusive treatment categories, including a no current treatment group and 20 categories for monotherapy, dual treatment, and triple combinations of drugs.

### Confounding variables

We considered confounding variables that were likely to be associated with the risk of the complications from diabetes^20^ ^37^ ^38^ ^39^ ^40^ or with the likelihood of receiving treatment with different diabetes drugs. These included age at study entry, sex, number of years since diabetes was diagnosed (<1 year and 1-3, 4-6, 7-10, and ≥ 11 years),^40^ calendar year, smoking status (non-smoker; former smoker; light smoker, 1-9 cigarettes/day; moderate smoker, 10-19 cigarettes/day; heavy smoker, ≥20 cigarettes/day; not recorded), ethnic group (white/not recorded, Indian, Pakistani, Bangladeshi, other Asian, black African, black Caribbean, Chinese, other, including mixed),^37^ Townsend deprivation score, previous diabetes complications (severe kidney failure,^40^ ≥1 episodes of hyperglycaemia, ≥1 episodes of hypoglycaemia, lower limb amputation, blindness), comorbidities (cardiovascular disease^40^ (other than when cardiovascular disease was the outcome of interest), heart failure (other than when heart failure was the outcome of interest), peripheral vascular disease, valvular heart disease, chronic kidney disease, atrial fibrillation,^37^ hypertension,^37^ rheumatoid arthritis^37^)), prescription drugs (statins, aspirin, anticoagulants, thiazides, angiotensin converting enzyme inhibitors, angiotensin receptor blockers, calcium channel blockers), and clinical values (body mass index kg/m,^2^ ^40^ cholesterol to high density lipoprotein cholesterol ratio,^37^ systolic blood pressure (mm Hg),^37^ serum creatinine level, glycated haemoglobin A1_c_ (mmol/mol).^40^ ^41^ We evaluated confounders at the start of each treatment period for comorbidities, previous complications, other prescribed drugs, smoking status, and clinical values. For comorbidities and previous complications, we identified whether patients had a diagnosis recorded before the relevant treatment period. For prescribed drugs, we defined patients as treated at the start of the relevant period of diabetes drug treatment if they had at least two prescriptions for the other type of drug, including one in the 28 days before the treatment period and one after the start date. For smoking status and continuous variables (systolic blood pressure, body mass index, creatinine level, cholesterol to high density lipoprotein cholesterol ratio, and haemoglobin A1_c_), we used the most recent recorded value immediately before the relevant treatment period.

### Statistical analysis

Using Cox proportional hazards models we assessed the associations between the six different classes of diabetes drugs and risk of each of our three outcomes, adjusting for potential confounding variables. Rather than using a competing risks analysis we used the Cox model as it is considered more appropriate for analyses of causes such as in this study, whereas competing risks analyses tend to be more useful for prediction modelling or estimating absolute risks.^42^ ^43^ ^44^ To account for patients starting and stopping different diabetes treatments and changing between treatments, we included use of different diabetes drugs as time varying exposures. In the analysis, we calculated unadjusted and adjusted hazard ratios for the six different classes of diabetes drug (each as a binary variable indicating use or no use), with adjustment for the confounding variables and the other classes of diabetes drugs. We also calculated unadjusted and adjusted hazard ratios for the mutually exclusive treatment combinations comparing each treatment category with no current treatment. To determine whether there were significant differences between classes or individual drugs, we carried out Wald’s tests. We tested for interactions between the six different drug classes and age, sex, haemoglobin A1_c_, and body mass index. We used multiple imputation with chained equations to replace missing values for continuous values and smoking status and used these values in our main analyses.^45^ ^46^ ^47^ We did this for each of the study outcomes and in the imputation model included the censoring indicator for the outcome, the log of survival time, all the confounding variables, and the diabetes drug treatment variables. Before imputation we log transformed body mass index, haemoglobin A1_c_, creatinine level, and cholesterol to high density lipoprotein cholesterol ratio, as they had skewed distributions. We carried out five imputations, and combined the results using Rubin’s rules.

To evaluate the robustness of our results and assess the impact of confounding variables we added the confounding variables to our model in blocks and compared the adjusted hazard ratios. Box 1 lists the models we assessed.

**Box 1: Types of models used in study**

- Model A: diabetes drug classes adjusted for age, sex, ethnicity, deprivation, calendar year, duration of diabetes, plus other diabetes drugs
- Model B: model A plus comorbidities (hypertension, cardiovascular disease, heart failure, atrial fibrillation, chronic kidney disease, rheumatoid arthritis, valvular heart disease, peripheral vascular disease) plus previous complications (hypoglycaemia, hyperglycaemia, amputation, severe kidney failure, blindness) plus use of other drugs (statins, aspirin, anticoagulants, thiazides, angiotensin converting enzyme inhibitors or angiotensin receptor blockers, calcium channel blockers)
- Model C (primary analysis model): model B plus clinical values (body mass index, cholesterol to high density lipoprotein cholesterol ratio, systolic blood pressure, serum creatinine level, haemoglobin A1_c_)
- Model D: model C plus interaction terms
- Model E: treatment combinations categorical variable plus confounders in model C (models compared with no treatment and with metformin monotherapy)
- Model F: model C with prevalent users of sulphonylureas excluded
- Model G: model E with prevalent users of sulphonylureas excluded

We also carried out a sensitivity analysis where we excluded prevalent users of sulphonylureas from the study cohort so that the hazard ratios for sulphonylureas are based on incident users, and we fitted the models F and G (see box 1).

We used all the available data in the database to maximise the power and generalisability of the results. P values less than 0.01 (two tailed) were considered as significant and hazard ratios of 1.10 or more or 0.90 or less as clinically important. STATA (version 13.1) was used for all analyses.

### Patient involvement

Patients were not involved in setting the research question, in the outcome measures, in the design, or in the implementation of the study. Patient representatives from the QResearch advisory board have written the information for patients on the QResearch website about the use of the database for research. They have also advised on dissemination, including the use of lay summaries describing the research and its results.

## Results

Overall, 1243 practices contributing to QResearch in England met the inclusion criteria. A cohort of 601 405 patients aged 25-84 years with diabetes was identified (fig 1). We sequentially excluded 31 224 people with type 1 diabetes (5.1%), 748 (0.1%) without a Townsend deprivation score, and 99 745 prescribed glitazones, gliptins, or insulin in the 12 months before the study entry date, leaving 469 688 patients with type 2 diabetes in the study cohort. Figure 1 also shows the numbers of patients with each outcome at baseline who were excluded from the analysis of that outcome, as well as the numbers of incident outcomes observed during follow-up.

**Figure f1:**
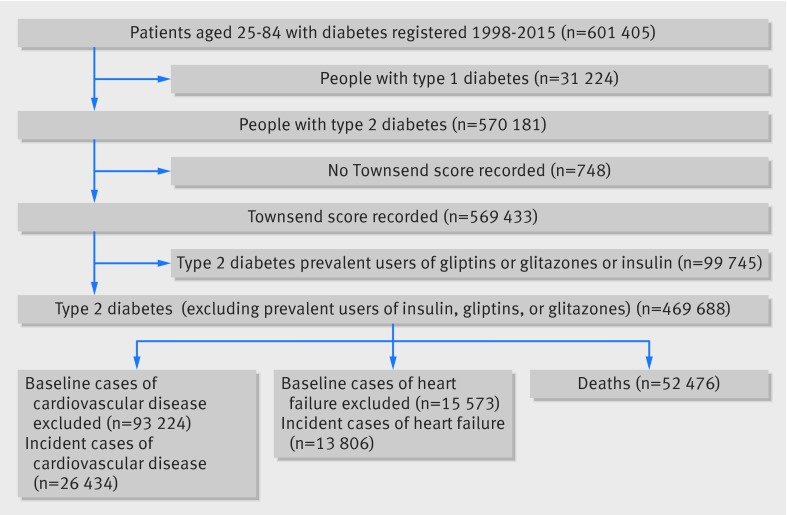
**Fig 1** Flow of people through study

### Baseline characteristics

In total 274 324 (58.4%) of the patients in the study cohort received prescriptions for one or more diabetes drugs during follow-up: 21 308 (4.5%) for glitazones, 32 533 (6.9%) for gliptins, 256 024 (54.5%) for metformin, 134 570 (28.7%) for sulphonylureas, 19 791 (4.2%) for insulin, and 12 062 (2.6%) for other oral diabetes drugs.

Table 1 shows the characteristics of patients who started each of the six classes of diabetes drugs during follow-up based on the last recorded value before the drug was first prescribed (or at study entry for patients already prescribed sulphonylureas, metformin, or other diabetes drugs at baseline). The groups were similar for most characteristics except for higher levels of comorbidities other than hypertension in patients prescribed insulin, and lower levels of prescriptions for statins and aspirin in patients prescribed metformin compared with the other drugs.

**Table 1 tbl1:** Characteristics of patients with type 2 diabetes when starting drugs or at study entry for prevalent users. Values are numbers (percentages) unless stated otherwise

Characteristics	Glitazones	Gliptins	Metformin	Sulphonylureas	Insulin	Other diabetes drugs
Total No of patients exposed	21 308	32 533	256 024	134 570	19 791	12 062
Median No of years exposed	4.5	5.7	4.8	4.9	5.9	4.9
Mean (SD) age at study entry	63.0 (11.9)	63.3 (12.1)	64.6 (13.1)	66.2 (12.9)	64.5 (12.7)	60.0 (11.9)
Mean (SD) Townsend score	0.4 (3.5)	0.5 (3.5)	0.6 (3.6)	0.6 (3.6)	0.5 (3.6)	0.8 (3.6)
Male	12 658 (59.4)	18 871 (58.0)	146 690 (57.3)	79 284 (58.9)	11 499 (58.1)	6509 (54.0)
Ethnicity:						
Ethnicity recorded	19 130 (89.8)	29 396 (90.4)	228 962 (89.4)	119 507 (88.8)	17 264 (87.2)	10 947 (90.8)
White or not recorded	17 112 (80.3)	26 104 (80.2)	204 915 (80.0)	107 537 (79.9)	17 001 (85.9)	10 135 (84.0)
Indian	997 (4.7)	1662 (5.1)	11 732 (4.6)	5978 (4.4)	476 (2.4)	420 (3.5)
Pakistani	811 (3.8)	1132 (3.5)	7425 (2.9)	3972 (3.0)	389 (2.0)	290 (2.4)
Bangladeshi	586 (2.8)	713 (2.2)	7282 (2.8)	3980 (3.0)	370 (1.9)	374 (3.1)
Other Asian	476 (2.2)	720 (2.2)	5873 (2.3)	2947 (2.2)	234 (1.2)	164 (1.4)
Caribbean	473 (2.2)	795 (2.4)	6376 (2.5)	3700 (2.7)	549 (2.8)	278 (2.3)
Black African	392 (1.8)	676 (2.1)	5715 (2.2)	2977 (2.2)	350 (1.8)	161 (1.3)
Chinese	84 (0.4)	95 (0.3)	983 (0.4)	513 (0.4)	36 (0.2)	34 (0.3)
Other	377 (1.8)	636 (2.0)	5723 (2.2)	2966 (2.2)	386 (2.0)	206 (1.7)
Smoking status:						
Smoking status recorded	21 215 (99.6)	32 399 (99.6)	255 186 (99.7)	134 080 (99.6)	19 569 (98.9)	12 003 (99.5)
Non-smoker	11 374 (53.4)	17 116 (52.6)	132 634 (51.8)	69 849 (51.9)	9393 (47.5)	6126 (50.8)
Former smoker	6252 (29.3)	9725 (29.9)	78 935 (30.8)	41 438 (30.8)	6142 (31.0)	3726 (30.9)
Light smoker	2170 (10.2)	3358 (10.3)	25 678 (10.0)	13 846 (10.3)	2413 (12.2)	1252 (10.4)
Moderate smoker	730 (3.4)	1121 (3.4)	9395 (3.7)	4661 (3.5)	832 (4.2)	441 (3.7)
Heavy smoker	689 (3.2)	1079 (3.3)	8544 (3.3)	4286 (3.2)	789 (4.0)	458 (3.8)
Comorbidities:						
Cardiovascular disease	2962 (13.9)	5325 (16.4)	48 066 (18.8)	28 895 (21.5)	4596 (23.2)	1992 (16.5)
Heart failure	302 (1.4)	737 (2.3)	6943 (2.7)	5069 (3.8)	960 (4.9)	374 (3.1)
Peripheral vascular disease	1008 (4.7)	1576 (4.8)	12458 (4.9)	8467 (6.3)	1519 (7.7)	544 (4.5)
Valvular heart disease	379 (1.8)	914 (2.8)	7378 (2.9)	4606 (3.4)	765 (3.9)	292 (2.4)
Hypertension	12 520 (58.8)	19 293 (59.3)	150 219 (58.7)	80 776 (60.0)	11 117 (56.2)	7310 (60.6)
Atrial fibrillation	929 (4.4)	1980 (6.1)	17327 (6.8)	10574 (7.9)	1890 (9.5)	657 (5.4)
Chronic kidney disease	388 (1.8)	593 (1.8)	3067 (1.2)	4183 (3.1)	1165 (5.9)	224 (1.9)
Rheumatoid arthritis	719 (3.4)	1237 (3.8)	9718 (3.8)	5382 (4.0)	842 (4.3)	460 (3.8)
Previous complications:						
Severe kidney disease	54 (0.3)	74 (0.2)	509 (0.2)	825 (0.6)	213 (1.1)	36 (0.3)
Blindness	260 (1.2)	383 (1.2)	3715 (1.5)	2404 (1.8)	360 (1.8)	170 (1.4)
Amputation	85 (0.4)	125 (0.4)	1239 (0.5)	894 (0.7)	161 (0.8)	65 (0.5)
≥1 previous episode of hypoglycaemia	288 (1.4)	286 (0.9)	2247 (0.9)	1946 (1.4)	337 (1.7)	215 (1.8)
≥1 previous episode of hyperglycaemia	7921 (37.2)	10 054 (30.9)	68 839 (26.9)	46 341 (34.4)	7279 (36.8)	3914 (32.4)
Other drugs:						
anticoagulant	642 (3.0)	1419 (4.4)	9409 (3.7)	5989 (4.5)	1344 (6.8)	540 (4.5)
Thiazides	3444 (16.2)	4346 (13.4)	31 291 (12.2)	16 972 (12.6)	2386 (12.1)	1844 (15.3)
ACE inhibitors	9318 (43.7)	12 939 (39.8)	83 847 (32.7)	48 960 (36.4)	7750 (39.2)	5362 (44.5)
Angiotension receptor blockers	3399 (16.0)	4895 (15.0)	28 629 (11.2)	16 976 (12.6)	2633 (13.3)	2088 (17.3)
Calcium channel blockers	5613 (26.3)	8105 (24.9)	55 674 (21.7)	32 141 (23.9)	5034 (25.4)	3328 (27.6)
Statins	15 512 (72.8)	21 383 (65.7)	137 574 (53.7)	77 865 (57.9)	12 640 (63.9)	8451 (70.1)
Aspirin	7890 (37.0)	9684 (29.8)	68 013 (26.6)	41 647 (30.9)	7057 (35.7)	4096 (34.0)

ACE=angiotensin converting enzyme.

Values represent those recorded before starting drugs or at study entry for prevalent users. Treatment groups not mutually exclusive.

Table 2 shows levels of recording and mean values for haemoglobin A1_c_, body mass index, cholesterol to high density lipoprotein cholesterol ratio, systolic blood pressure, and serum creatinine level before starting treatment. The highest levels of recording were for haemoglobin A1_c_, which were in excess of 97% for all six drug groups. Lowest levels of recording were for cholesterol to high density lipoprotein cholesterol ratios, which were more than 84% for all drug groups. Overall, at least 82% of patients had complete data for each clinical value across all drug groups. Apart from higher mean levels of haemoglobin A1_c_ in patients before use of insulin or the group of other diabetes drugs, and higher levels of creatinine among those prescribed sulphonylureas or insulin, the mean values were similar across the six groups. Supplementary table 1 shows mean values before starting the 20 different treatment combinations. The mean values for haemoglobin A1_c_ tended to be higher for patients starting triple treatment (as high values tend to trigger changes in treatment).

**Table 2 tbl2:** Recorded clinical values before starting drugs or at study entry for prevalent users

Clinical variables	Glitazones	Gliptins	Metformin	Sulphonylureas	Insulin	Other diabetes drugs
Total No of patients exposed	21 308	32 533	256 024	134 570	19 791	12 062
Variables recorded (%):						
Haemoglobin A1_c_	21 251 (99.7)	32 474 (99.8)	253 219 (98.9)	133 170 (99.0)	19 255 (97.3)	12 022 (99.7)
Body mass index	21 120 (99.1)	32 224 (99.1)	252 290 (98.5)	132 477 (98.4)	19 436 (98.2)	11 749 (97.4)
Cholesterol to HDL ratio	18 264 (85.7)	29 307 (90.1)	224 504 (87.7)	115 991 (86.2)	16 723 (84.5)	10 619 (88.0)
Systolic blood pressure	21 306 (100.0)	32 529 (100.0)	255 892 (99.9)	134 487 (99.9)	19 765 (99.9)	12 057 (100.0)
Creatinine level	21 288 (99.9)	32 509 (99.9)	255 381 (99.7)	134 244 (99.8)	19 655 (99.3)	12 044 (99.9)
Total recorded	18 097 (84.9)	29 010 (89.2)	220 119 (86.0)	113 843 (84.6)	16 270 (82.2)	10 340 (85.7)
Mean (SD) values:						
Haemoglobin A1_c_ (mmol/mol)	66.8 (18.9)	68.4 (18.4)	61.4 (18.7)	64.9 (19.9)	75.4 (22.7)	70.9 (19.6)
Body mass index (kg/m^2^)	31.7 (6.0)	31.7 (5.9)	30.6 (5.9)	30.1 (5.8)	30.2 (6.1)	34.1 (6.6)
Cholesterol to HDL ratio	3.8 (1.3)	3.9 (1.3)	3.8 (1.3)	3.8 (1.3)	4.0 (1.4)	4.0 (1.3)
Systolic blood pressure (mm Hg)	133.1 (14.8)	132.3 (14.7)	132.5 (15.3)	132.8 (15.9)	131.7 (16.9)	132.5 (14.9)
Creatinine level	87.1 (33.7)	84.9 (33.3)	84.8 (30.1)	92.1 (47.7)	99.3 (62.0)	83.5 (34.6)

HDL=high density lipoprotein cholesterol.

Values represent those recorded before starting drugs or at study entry for prevalent users. Treatment groups not mutually exclusive.

### Risks associated with use of each diabetes drug group

Table 3 shows the number of incident cases of each outcome for patients’ periods of use of each of the six treatment groups during follow-up. Importantly, the treatment groups in table 3 are not mutually exclusive—for example, the rows for glitazones include any use of glitazones, whether as monotherapy, dual treatment, or triple treatment. Similarly, the adjusted hazard ratios for model C shown in table 3 give an overall risk for the use of each drug group compared with non-use of that drug group, having adjusted for use of other diabetes drugs and the potential confounders listed in the footnote.

**Table 3 tbl3:** Adjusted hazard ratio (95% confidence intervals) for each outcome by use of diabetes drug (model C)

Outcomes and drugs	No of cases	Person years	Rate per 10 000	Adjusted hazard ratio (95% CI)
All cause mortality:				
Glitazones	597	55 916	106.8	0.77 (0.71 to 0.84)*
Gliptins	996	71 524	139.3	0.82 (0.77 to 0.88)*
Metformin	17 109	1 066 516	160.4	0.59 (0.58 to 0.60)*
Sulphonylureas	12 717	506 719	251.0	1.10 (1.07 to 1.12)†
Insulin	2529	57 875	437.0	1.47 (1.41 to 1.53)†
Other diabetes drugs	350	28 293	123.7	0.82 (0.73 to 0.91)*
Heart failure:				
Glitazones	308	55 051	56.0	0.74 (0.66 to 0.83)*
Gliptins	421	68 724	61.3	0.86 (0.78 to 0.95)*
Metformin	6785	1 029 331	65.9	0.70 (0.68 to 0.73)*
Sulphonylureas	4415	481 339	91.7	1.04 (1.00 to 1.08)‡
Insulin	686	52 634	130.3	1.32 (1.22 to 1.43)†
Other diabetes drugs	192	26 950	71.2	0.92 (0.79 to 1.06)‡
Cardiovascular disease:				
Glitazones	699	47 374	147.6	0.75 (0.69 to 0.81)*
Gliptins	912	56 414	161.7	0.94 (0.88 to 1.00)‡
Metformin	14 218	834 914	170.3	0.76 (0.74 to 0.78)*
Sulphonylureas	7999	378 816	211.2	1.00 (0.97 to 1.03)‡
Insulin	985	39 870	247.1	1.23 (1.15 to 1.31)†
Other diabetes drugs	396	22 152	178.8	0.95 (0.86 to 1.05)‡

Hazard ratios adjusted for: sex; age; calendar year; duration since diagnosis of diabetes (five levels); ethnicity (nine levels); Townsend deprivation score; smoking status (five levels); use of other diabetes drugs, anticoagulants, thiazides, angiotensin converting enzyme inhibitors, angiotensin receptor blockers, calcium channel blockers, statins, or aspirin, previous complications (blindness, hyperglycaemia, hypoglycaemia, amputation, severe kidney failure); hypertension; cardiovascular disease; atrial fibrillation; chronic renal disease; rheumatoid arthritis; valvular heart disease; peripheral vascular disease; body mass index; systolic blood pressure; haemoglobin A1_c_; serum creatinine level; cholesterol to high density lipoprotein ratio.

*Significantly decreased hazard ratio.

†Significantly increased hazard ratio.

‡Non-significant results.

For our main exposures of interest we found that compared with non-use, glitazones were significantly associated with a 23% decreased risk of all cause mortality, a 26% decreased risk of heart failure, and a 25% decreased risk of cardiovascular disease; and compared with non-use, gliptins were significantly associated with an 18% decreased risk of all cause mortality, a 14% decreased risk of heart failure, and no significant association with risk of cardiovascular disease.

In addition, for the other diabetes drug groups we found that compared with non-use, metformin was associated with a significantly decreased risk of all three outcomes—41% decreased risk of all cause mortality, a 30% decreased risk of heart failure, and a 24% decreased risk of cardiovascular disease; compared with non-use, sulphonylureas were significantly associated with a 10% increased risk of all cause mortality; compared with non-use, insulin was associated with a 47% increased risk of all cause mortality, a 32% increased risk of heart failure, and a 23% increased risk of cardiovascular disease; and compared with non-use, the other diabetes drugs group was significantly associated with an 18% decreased risk of all cause mortality.

For all cause mortality, we found significant interactions between glitazones and both age and haemoglobin A1_c_ (see supplementary tables 2a and b; model D) where the reduced risk of all cause mortality associated with glitazone use became less marked with both increasing age and increasing levels of haemoglobin A1_c_. Similarly, for cardiovascular disease, there were significant interactions between glitazones and haemoglobin A1_c_, where the reduced risk of cardiovascular disease associated with glitazone use became less marked with increasing levels of haemoglobin A1_c_.

We found similar interactions in both magnitude and direction for gliptins and both age and haemoglobin A1_c_ for all cause mortality and between gliptins and haemoglobin A1_c_ for cardiovascular disease.

Supplementary table 2 also shows the results from analyses with confounders added in separate blocks (models A and B). Generally, including comorbidities and previous complications in the model (model B) tended to result in similar hazard ratios for gliptins and glitazones (compared with model A). Further inclusion of clinical values (table 3: model C) only resulted in small changes to the hazard ratios. The sensitivity analysis excluding prevalent users of sulphonylureas at study entry showed increases in adjusted hazard ratios for all cause mortality and cardiovascular disease for insulin (see supplementary table 2: model E compared with model C) but similar hazard ratios for the other diabetes drug groups for all three outcomes.

### Risks associated with different treatment combinations

Table 4 provides a more detailed breakdown of 21 mutually exclusive treatment categories, including a no current treatment group, which included 0.7 million person years free of use of any diabetes drug. The table shows the number of events for each clinical outcome for each of the treatment categories.

**Table 4 tbl4:** Number of incident events for each outcome and person years of exposure to mutually exclusive treatment groups

Variables	Heart failure			Cardiovascular disease			All cause mortality	
	No of cases	Person years		No of cases	Person years		No of cases	Person years
Periods with no treatment	5317	675 598		9769	541 445		27 367	707 183
Monotherapy:								
Metformin	3334	570 230		7376	464 698		9815	589 353
Sulphonylureas	1211	82 466		1752	59 918		5654	90 846
Insulin	220	14 802		290	11 408		1349	16 859
Glitazones	9	1671		24	1352		48	1704
Gliptins	23	2361		43	1874		112	2560
Other diabetes drugs	18	1474		31	1168		72	1618
Dual treatment:								
Metformin and sulphonylureas	2439	304 023		4772	242 136		5350	316 576
Metformin and insulin	189	17 328		262	13 153		399	18 540
Metformin and glitazones	75	17 825		160	15 742		163	18 024
Metformin and gliptins	114	23 530		263	19 898		257	24 218
Metformin and other diabetes drugs	60	9009		119	7,479		85	9375
Sulphonylureas and insulin	94	3756		100	2558		369	4593
Sulphonylureas and glitazones	37	3444		59	2691		114	3564
Sulphonylureas and gliptins	47	4068		71	2988		164	4451
Sulphonylureas and other diabetes drugs	21	973		22	765		46	1096
Triple treatment:								
Metformin, sulphonylureas, and insulin	123	10 893		212	8209		283	11 593
Metformin, sulphonylureas, and glitazones	133	24 790		344	21 295		209	25 168
Metformin, sulphonylureas, and gliptins	181	30 617		412	24 885		355	31 762
Metformin, sulphonylureas, and other	60	9636		127	7902		87	10 070
All other drug combinations	101	13 519		226	11 160		178	14 127
Total	13 806	1 822 013		26 434	1 462 724		52 476	1 903 280

Table 5 shows the corresponding adjusted hazard ratios for each treatment group category compared with no current treatment. The key significant findings are (all compared with periods of no diabetes drug treatment):

**Table 5 tbl5:** Adjusted hazard ratio (95% confidence intervals) for each outcome (model F)

Outcomes by treatment	Adjusted hazard ratio (95% CI)		
	Heart failure	Cardiovascular disease	All cause mortality
No current treatment (reference)	1.00	1.00	1.00
Monotherapy:			
Metformin	0.68 (0.65 to 0.71)*	0.76 (0.74 to 0.79*	0.64 (0.63 to 0.66*
Sulphonylureas	1.00 (0.94 to 1.07)‡	1.00 (0.95 to 1.05)‡	1.24 (1.20 to 1.28)†
Insulin	1.26 (1.10 to 1.44)†	1.22 (1.08 to 1.37)†	1.64 (1.55 to 1.74)†
Glitazones	0.50 (0.26 to 0.97*	0.79 (0.53 to 1.18)‡	0.89 (0.67 to 1.18)‡
Gliptins	0.87 (0.58 to 1.31)‡	1.14 (0.85 to 1.54)‡	1.20 (1.00 to 1.44)‡
Other diabetes drugs	0.92 (0.58 to 1.46)‡	1.12 (0.79 to 1.59)‡	1.06 (0.84 to 1.33)‡
Dual treatment:			
Metformin and sulphonylureas	0.74 (0.70 to 0.78)*	0.75 (0.73 to 0.78)*	0.62 (0.60 to 0.64)*
Metformin and insulin	1.08 (0.93 to 1.25)‡	0.89 (0.78 to 1.01)‡	0.76 (0.69 to 0.84)*
Metformin and glitazones	0.50 (0.40 to 0.63)*	0.46 (0.39 to 0.54)*	0.55 (0.47 to 0.64)*
Metformin and gliptins	0.62 (0.52 to 0.75)*	0.67 (0.59 to 0.75)*	0.52 (0.46 to 0.59)*
Metformin and other diabetes drugs	0.74 (0.57 to 0.95)*	0.73 (0.61 to 0.88)*	0.46 (0.37 to 0.57)*
Sulphonylureas and insulin	1.18 (0.96 to 1.45)‡	1.18 (0.96 to 1.44)‡	1.49 (1.35 to 1.66)†
Sulphonylureas and glitazones	0.65 (0.47 to 0.89)*	0.75 (0.58 to 0.98)*	0.96 (0.80 to 1.16)‡
Sulphonylureas and gliptins	0.88 (0.66 to 1.17)‡	0.97 (0.76 to 1.22)‡	0.92 (0.79 to 1.08)‡
Sulphonylureas and other diabetes drugs	1.46 (0.95 to 2.24)‡	1.02 (0.67 to 1.55)‡	1.33 (1.00 to 1.78)‡
Triple treatment:			
Metformin, sulphonylureas, and insulin	0.91 (0.76 to 1.09)‡	0.95 (0.82 to 1.09)‡	0.98 (0.87 to 1.10)‡
Metformin, sulphonylureas, and glitazones	0.54 (0.45 to 0.64)*	0.59 (0.53 to 0.66)*	0.44 (0.38 to 0.50)*
Metformin, sulphonylureas, and gliptins	0.60 (0.52 to 0.70)*	0.70 (0.63 to 0.78)*	0.49 (0.44 to 0.55)*
Metformin, sulphonylureas, and other diabetes drugs	0.60 (0.46 to 0.77)*	0.62 (0.52 to 0.74)*	0.46 (0.37 to 0.57)*
All other drug combinations	0.72 (0.59 to 0.88)*	0.82 (0.72 to 0.94)*	0.68 (0.58 to 0.79)*

*Significantly decreased.

†Significantly increased.

‡Non-significant.

Hazard ratios adjusted for: sex; age; calendar year; duration since diagnosis of diabetes (five levels); ethnicity (nine levels); Townsend deprivation score; smoking status (five levels); use of other diabetes drugs, anticoagulant thiazides, angiotensin coverting enzyme inhibitors, angiotensin receptor blockers, calcium channel blockers, statins, and aspirin; previous complications (blindness, hyperglycaemia, hypoglycaemia, amputation, severe kidney failure); hypertension; cardiovascular disease; atrial fibrillation; chronic renal disease; rheumatoid arthritis; valvular heart disease; peripheral vascular disease; body mass index; systolic blood pressure; haemoglobin A1_c_; serum creatinine level; cholesterol to high density lipoprotein cholesterol ratio.

• There were no significant associations between monotherapy with gliptins and risk of any of the three outcomes. Dual treatment with gliptins and metformin was associated with a decreased risk of all three outcomes (reductions of 38% for heart failure, 33% for cardiovascular disease, and 48% for all cause mortality). Triple treatment with metformin, sulphonylureas, and gliptins was associated with a decreased risk of all three outcomes (reductions of 40% for heart failure, 30% for cardiovascular disease, and 51% for all cause mortality).

• Monotherapy with glitazones was associated with a 50% decreased risk of heart failure. Dual treatment with metformin and glitazones was associated with a decreased risk of all three outcomes (reductions of 50% for heart failure, 54% for cardiovascular disease, and 45% for all cause mortality). Dual treatment with sulphonylureas and glitazones was associated with a decreased risk of two outcomes (reductions of 35% for heart failure and 25% for cardiovascular disease). Triple treatment with metformin, sulphonylureas, and glitazones was associated with a decreased risk of all three outcomes (reductions of 46% for heart failure, 41% for cardiovascular disease, and 56% for all cause mortality).

Table 6 shows the results for the different treatment combinations compared with metformin treatment alone (rather than compared with periods of no treatment as previously). The key findings for our two exposures of interest are:

**Table 6 tbl6:** Adjusted hazard ratios (95% confidence intervals) for each outcome (model F) compared with monotherapy with metformin

Treatments	Adjusted hazard ratio (95% CI)		
	Heart failure	Cardiovascular disease	All cause mortality
Metformin (reference)	1.00	1.00	1.00
Monotherapy or no treatment:			
No current treatment	1.47 (1.40 to 1.53)*	1.31 (1.27 to 1.35)*	1.55 (1.52 to 1.59)*
Sulphonylureas	1.47 (1.37 to 1.57)*	1.31 (1.24 to 1.38)*	1.93 (1.87 to 2.00)*
Insulin	1.85 (1.61 to 2.12)*	1.59 (1.41 to 1.80)*	2.55 (2.41 to 2.71)*
Glitazones	0.74 (0.38 to 1.42)†	1.03 (0.69 to 1.54)†	1.38 (1.04 to 1.83)†
Gliptins	1.28 (0.85 to 1.92)†	1.50 (1.11 to 2.02)*	1.86 (1.55 to 2.25)*
Other diabetes drugs	1.35 (0.85 to 2.14)†	1.47 (1.03 to 2.09)*	1.65 (1.31 to 2.08)*
Dual treatment:			
Metformin and sulphonylureas	1.09 (1.03 to 1.15)*	0.99 (0.95 to 1.03)†	0.96 (0.93 to 0.99)†
Metformin and insulin	1.58 (1.36 to 1.83)*	1.16 (1.03 to 1.32)*	1.18 (1.07 to 1.31)*
Metformin and glitazones	0.74 (0.58 to 0.93)‡	0.60 (0.51 to 0.70)‡	0.86 (0.74 to 1.00)†
Metformin and gliptins	0.91 (0.76 to 1.10)†	0.87 (0.77 to 0.99)‡	0.80 (0.71 to 0.91)‡
Metformin and other hypo	1.08 (0.84 to 1.40)†	0.96 (0.80 to 1.15)†	0.72 (0.58 to 0.89)‡
Sulphonylureas and insulin	1.73 (1.41 to 2.13)*	1.54 (1.26 to 1.88)*	2.32 (2.09 to 2.58)*
Sulphonylureas and glitazones	0.95 (0.68 to 1.31)†	0.99 (0.76 to 1.28)†	1.50 (1.24 to 1.80)*
Sulphonylureas and gliptins	1.29 (0.96 to 1.72)†	1.27 (1.00 to 1.60)†	1.44 (1.23 to 1.68)*
Sulphonylureas and other diabetes drugs	2.13 (1.39 to 3.28)*	1.33 (0.87 to 2.03)†	2.07 (1.55 to 2.77)*
Triple treatment:			
Metformin, sulphonylureas, and insulin	1.34 (1.11 to 1.60)*	1.24 (1.08 to 1.42)*	1.53 (1.35 to 1.72)*
Metformin, sulphonylureas, and glitazones	0.79 (0.66 to 0.93)‡	0.78 (0.70 to 0.87)‡	0.68 (0.59 to 0.78)‡
Metformin, sulphonylureas, and gliptins	0.89 (0.76 to 1.03)†	0.92 (0.83 to 1.02)†	0.76 (0.69 to 0.85)‡
Metformin, sulphonylureas, and other diabetes drugs	0.87 (0.68 to 1.13)†	0.81 (0.68 to 0.97)‡	0.71 (0.58 to 0.88)‡
All other drug combinations	1.06 (0.86 to 1.29)†	1.07 (0.94 to 1.23)†	1.05 (0.91 to 1.22)†

*Significantly increased.

†Non-significant.

‡Significantly decreased.

Hazard ratios adjusted for: sex; age; calendar year; duration since diagnosis of diabetes (five levels); ethnicity (nine levels); Townsend deprivation score; smoking status (five levels); use of other diabetes drugs, anticoagulant thiazides, angiotensin coverting enzyme inhibitors, angiotensin receptor blockers, calcium channel blockers, statins, and aspirin; previous complications (blindness, hyperglycaemia, hypoglycaemia, amputation, severe kidney failure); hypertension; cardiovascular disease; atrial fibrillation; chronic renal disease; rheumatoid arthritis; valvular heart disease; peripheral vascular disease; body mass index; systolic blood pressure; haemoglobin A1_c_; serum creatinine level; cholesterol to high density lipoprotein cholesterol ratio.

• Monotherapy with gliptins was associated with a 50% increased risk of cardiovascular disease and an 86% increased risk of all cause mortality; dual treatment with metformin and gliptins was associated with a decreased risk of two outcomes (reductions of 13% for cardiovascular disease and 20% for all cause mortality); dual treatment with sulphonylureas and gliptins was associated with a 44% increased risk of all cause mortality and a borderline 27% increased risk of cardiovascular disease; triple treatment with metformin, sulphonylureas, and gliptins was associated with a 24% decreased risk of all cause mortality compared with metformin alone.

• Monotherapy with glitazones was not associated with an increased or decreased risk of any outcome compared with metformin alone; dual treatment with metformin and glitazones was associated with a decreased risk of two outcomes (reductions of 26% for heart failure and 40% for cardiovascular disease) and a borderline 14% decreased risk in all cause mortality; dual treatment with sulphonylureas and glitazones was associated with a 50% increased risk of all cause mortality; triple treatment with metformin, sulphonylureas, and glitazones was associated with a decreased risk of all three outcomes (reductions of 21% for heart failure, 22% for cardiovascular disease, and 32% for all cause mortality) compared with metformin alone.

Supplementary table 3 shows the results for the different treatment combinations compared with periods of no treatment, having dropped prevalent users of sulphonylureas at study entry (model G). Incident use of sulphonlyureas, whether alone or in combination with insulin as dual or triple treatment, was associated with a substantial increased risk of all cause mortality of 50%, 171%, and 87%, respectively. Monotherapy with sulphonylureas was associated with a 12% increased risk of cardiovascular disease and a 16% increased risk of heart failure. Triple treatment with sulphonlyureas, metformin, and insulin was associated with a 42% increased risk of heart failure. Overall, the adjusted hazard ratios for glitazones and gliptins were similar to those in table 5, except confidence intervals were wider, and there was no longer a significant decrease in risk of heart failure and cardiovascular disease among people prescribed dual treatment with sulphonylureas and glitazones.

## Discussion

We have conducted a large cohort study of risks for cardiovascular disease, heart failure, and all cause mortality associated with different diabetes drugs in people with type 2 diabetes attending for routine clinical care in the UK. Our companion paper^27^ presented the corresponding analysis for other complications of diabetes, including blindness, amputation, severe kidney failure, hyperglycaemia, and hypoglycaemia. The approach we have taken allows assessment of the relative benefits and hazards of diabetes drugs in a real world clinical setting for a range of clinically important outcomes. It enables analysis of treatment periods that are substantially longer than those reported in clinical trials,^9^ including more events than in previous similar observational studies.^5^ ^12^

We have found clinically relevant differences between different diabetes drugs (alone and in combination) in the risk of three key outcomes—heart failure, cardiovascular disease, and death—in people with type 2 diabetes. Compared with non-use, use of glitazones was significantly associated with a decreased risk of all three outcomes (all cause mortality; heart failure, and cardiovascular disease), whereas use of gliptins was significantly associated with a decreased risk of two outcomes (all cause mortality and heart failure) but no significant change in risk of cardiovascular disease. Compared with periods of no treatment, dual treatment (ie, metformin and gliptins or metformin and glitazones) was associated with a decreased risk of all three outcomes, as was triple treatment with metformin, sulphonylureas, and either gliptins or glitazones.

However, since the use of gliptins and glitazones is usually recommended as a second line treatment in combination with other agents such as metformin, the clinical question is whether the addition of gliptins or glitazones to metformin monotherapy is associated with net benefit or net harm. Compared with metformin monotherapy, dual treatment (metformin and glitazones) and triple treatment (metformin, sulphonylureas, and glitazones) were associated with decreased risk of all three outcomes (see table 6). Dual treatment (metformin and gliptins) was associated with a decreased risk of cardiovascular disease and all cause mortality compared with metformin monotherapy. Triple treatment (metformin, sulphonylureas, and gliptins) was associated with a 24% reduced risk of all cause mortality (see table 6).

### Gliptins

Our study included over 70 000 person years of exposure to gliptins from over 32 500 patients, which represents one of the largest studies to date, with over four times more gliptin users than in the observational study by Eurich et al.^20^ Our results differ from those reported by Eurich et al, since we found that use of gliptins compared with non-use was associated with a reduction in all cause mortality. We also found that dual treatment (metformin and gliptins) and triple treatment (metformin, sulphonylureas, and gliptins) seems to be associated with a lower risk of all cause mortality compared with monotherapy with metformin. The median duration of use of gliptins in our study was 5.7 years, compared with 2.5 years in the study by Eurich et al,^20^ and the mean age of our cohort was 63 years compared with 52 years.^20^ We also had more events and more statistical power—there were 996 deaths during 71 524 person years of exposure to gliptins in our study compared with 32 deaths during 11 307 person years in the study by Eurich et al.^20^ Our findings of a reduction in risk of all cause mortality are relatively novel and deserve further investigation, especially as there was no overall reduction in cardiovascular events. Our results are consistent with another recent real world observational study, which found gliptins associated with reduced all cause mortality.^48^ Park et al hypothesised that their findings might reflect reduced hypoglycaemic events, although their study, like ours, was observational and susceptible to unmeasured confounding.^48^ In our companion paper, we reported reduced risks of hypoglycaemia among gliptin users compared with non-users, which is broadly consistent with this hypothesis.^27^

Our results are also broadly consistent with recent non-inferiority trials^18^ and meta-analyses of randomised controlled trials, reporting that various gliptins (alogliptin, dutogliptin, linagliptin, saxagliptin, sitagliptin, and vildagliptin) are associated with statistically significant 30-60% reductions in major adverse cardiac events and non-significant 33% and 48% reductions in all cause and cardiovascular death compared with other active drugs or placebo treatment.^49^ ^50^ However, Monami et al urge caution in interpreting the results because the events were not the principle endpoints, the trial duration was short, and the characteristics of patients could be different from those of patients in routine clinical practice.^50^ We found an inverse association for gliptins, with a 15% lower risk of heart failure, whereas trials did not find such a strong benefit. This may reflect residual confounding owing to the observational study design or differences in study duration. However, our findings are consistent with the recently published multicentre nested case-control study,^51^ which also reported a 14-18% lower risk of hospital admission for heart failure with gliptins compared with other diabetes drugs. It is also consistent with a new user cohort study, which did not find a higher risk of heart failure among patients prescribed gliptins compared with other diabetes drugs.^52^

Some of the differences in results between our observational study and earlier clinical trials may reflect the type of gliptin studied—80% of patients prescribed gliptins were prescribed sitagliptin in our study compared with saxagliptin in some of the trials. Currently there are too few patients prescribed linagliptin, saxagliptin, and vildagliptin to support separate analyses by individual drug, which is a limitation of the study as there may be differences between individual gliptins and their effect on haemoglobin A1_c_.^53^ However, the numbers of people taking different types of gliptin is likely to increase over time, and further analyses can be undertaken once more data have accrued.

### Glitazones

Our study included over 55 000 person years of exposure to glitazones arising from 21 308 patients. The predominant glitazone was pioglitazone, which was prescribed to 90% of glitazone users, and hence our results most closely reflect associations for pioglitazone rather than rosiglitazone (withdrawn in the UK in 2010). Our study is substantially larger than a previous UK study of 92 000 people with diabetes, which finished in 2005^4^ and includes newer drugs available over the past decade. It supplements information from the Canadian study,^12^ of people aged 66 and over which compared three outcomes (heart failure, myocardial infarction, and death) among 16 951 users of pioglitazone compared with 22 785 users of rosiglitazone between 2002 and 2008. Our study is more recent and includes more patients prescribed pioglitazone over a longer duration (mean exposure of 4.5 years in our study compared with 294 days). It also includes more events—the Canadian study^12^ included 461 cases of heart failure, 273 of myocardial infarction, and 377 deaths.

We have found significant reductions in two outcomes—cardiovascular disease and all cause mortality—among users of glitazones compared with non-users (table 3). This reduction is similar in magnitude to that reported in clinical trials and meta-analyses of pioglitazone.^9^ ^11^ In 2007, Lincoff et al reported a meta-analysis of 19 trials of pioglitazone with a treatment duration ranging from four months to 3.5 years.^11^ The results showed an 18% decreased risk of their composite primary outcome (risk of death, myocardial infarction, or stroke). However, we have reported a decreased risk of heart failure among users of glitazones compared with non-users. This contrasts with the increase in heart failure that occurred in other studies, without an associated increase in mortality.^9^ ^11^ The authors of the PROactive study partially attributed this finding to a diagnostic bias due to the increase of oedema in the pioglitazone group.^9^ Our observational study differs from the clinical trial not only in design, size, and setting but also in the calendar time during which it was conducted and in the selection of patients. For example, the PROactive study^9^ selectively recruited high risk patients with pre-existing macrovascular disease, whereas our study included a more representative population from primary care. Two thirds of patients in the PROactive study^9^ had evidence of myocardial infarction or stroke compared with 14% in our study. It is also possible that, given the growing concerns about rosiglitazone and its subsequent withdrawal, patients with symptoms or signs suggestive of early heart failure (such as breathlessness or oedema) in our study might not have been prescribed pioglitazone or might have switched to another diabetes drug.

### Other diabetes drugs

Although our research question focuses on associations between gliptins and glitazones and adverse clinical outcomes, for comparison we have results for other diabetes drugs. Overall, metformin monotherapy was associated with reduced risks of all cause mortality, heart failure, and cardiovascular disease compared with non-use. This is important because metformin is generally recommended as the first line diabetes drug and is commonly used in combinations with other drugs. Sulphonylureas were associated with an increased risk of all cause mortality both in the main analysis and in the sensitivity analyses restricted to new users of sulphonylureas. Monotherapy with sulphonylureas was also associated with an increased risk of cardiovascular disease and as triple treatment with metformin and insulin was associated with an increased risk of heart failure compared with metformin alone. These unfavourable results are consistent with some studies linking sulphonylureas with increased adverse cardiovascular events,^5^ but not all studies.^26^ Further research into the safety of sulphonylureas compared with other types of diabetes drugs is warranted. However, this research needs to distinguish between individual types of sulphonylureas, as different individual drugs have different mortality risks.^54^

As in our companion paper,^27^ we excluded prevalent users of insulin at baseline but included in the analysis those patients subsequently prescribed insulin because insulin is part of the treatment ladder and some of these people will also have had other drugs of interest during follow-up. Although insulin was not the primary drug of interest, patients prescribed insulin had higher risks of all three outcomes, despite adjustment for higher levels of comorbidity. It is unlikely that this increased risk was a direct result of treatment with insulin. Instead, residual confounding and reverse causality could have occurred—that is, the insulin treated group was at much higher risk of complications than the groups treated with diet or oral drugs, and these result in their apparently worse outcomes and not their treatment with insulin. For example, in table 2, the insulin treated group had the highest haemoglobin A1_c_ and creatinine values before treatment, although both factors were adjusted for in the analyses. An alternative explanation could be that patients with symptoms that indicated cardiovascular disease or heart failure were prescribed insulin rather than glitazones or gliptins before subsequently having a diagnosis of these conditions.

### Strengths and limitations of this study

#### Generalisability

This is a large study based on an ethnically diverse contemporaneous, representative population of people with type 2 diabetes during an eight year study period. We included all eligible patients to minimise selection bias. Hence we think the results are likely to be generalisable to similar populations of people with type 2 diabetes. Although our observational study has limitations inherent in its design, it also has advantages over meta-analyses of clinical trials as these tend to be limited by ascertainment and reporting of events, short follow-up, lack of time to event data, and insufficient power to report on rare cardiovascular events.^23^

#### Clinical outcomes

Strengths of our analysis are the inclusion of hard clinical endpoints of cardiovascular disease, heart failure, and death based on clinical diagnoses recorded on at least one of three linked electronic data sources. Use of all three linked data sources was designed to minimise under-ascertainment of outcomes, which would otherwise lead to under-estimation of absolute risks. The cardiovascular disease and heart failure outcomes are based on clinical diagnoses made by the treating clinician rather than formally adjudicated events as would occur in a clinical trial. Although it is possible that some patients were recorded as having heart failure or cardiovascular disease who did not have this condition, such misclassification will not have affected the mortality outcome. UK general practices have good levels of accuracy and completeness in recording clinical diagnoses and prescribed drugs.^55^ Also, the diagnostic validity of such diagnoses in general practice has been shown to be high.^56^ Possible ascertainment bias of outcomes is unlikely to vary according to the type of diabetes drug prescribed so would not explain the associations we found.

#### Exposure to diabetes drugs

We had detailed information on use of diabetes drugs prescribed throughout the follow-up period, enabling us to develop a detailed categorisation of drug exposure time into 21 different treatment groups, including combinations of treatments. Recording of prescriptions issued in UK general practices has high levels of completeness.^57^ We ascertained patient characteristics, concurrent drugs, clinical values, and diagnoses at the beginning of each treatment period. This enabled us to account for switching between different treatments or treatment combinations, making adjustments for a large number of potential confounders. We undertook a time varying analysis, which analysed different types of monotherapy, dual treatment, and triple treatment over the study period. This reflects real world prescribing patterns over a long duration, allowing multiple comparisons not only between drugs but also between different combinations of drugs compared with untreated periods of time (diet only treatment). Our study analysed prescribed drugs rather than the drug actually taken by the patients, although renewal of prescriptions is likely to indicate drug use, as patients need to initiate repeat prescriptions. This could result in misclassification of exposure if patients were prescribed drugs that they did not actually take and could underestimate associations between use of diabetes drugs and clinical outcomes. Unlike previous studies, we have included comparisons of risk against periods of no treatment,^5^ which is important as about 40% of people with type 2 diabetes are managed without diabetes drugs throughout follow-up. Limitations include lack of analyses for different subtypes of each type of drug and for different dose levels.

#### Assessment of other types of bias

Other types of bias that can affect observational studies include recall bias, indication bias, and channelling bias. Recall bias will not have occurred as data on prescriptions for diabetes drugs and confounding variables were recorded before the clinical outcomes. We restricted the study population to people with type 2 diabetes to limit indication bias (ie, bias that occurs when people are prescribed drugs for a condition that is itself associated with the risk of the adverse event under consideration). We used an incident user design to reduce, but not eliminate, confounding and biases that can otherwise arise from adjustment for intermediate characteristics in the causal path.^36^ There were some differences at baseline between patients prescribed different treatment groups (tables 1 and 2[Table tbl1 tbl2]), although these were predominantly increased levels of comorbidities for insulin and lower levels of concurrent use of drugs (such as statins and aspirin) for metformin. To reduce channelling bias (where the choice of a particular drug is influenced by patient characteristics), we adjusted for a wide range of potential confounding variables. This included demographic characteristics (age, sex, ethnicity, and deprivation), different comorbidities, clinical values (including haemoglobin A1_c_, body mass index, blood pressure, creatinine level, and cholesterol to high density lipoprotein cholesterol ratio), and concurrent drugs. However, we are unable to exclude the possibility of residual confounding because other unmeasured patient characteristics might have affected the selection of diabetes drug treatment.

Although randomised controlled trials of diabetes drugs are not influenced by residual confounding, they tend to be small, of short duration, and might not report on relevant clinical outcomes. An alternative design would be an observational study of a cohort of patients specifically assembled for the purpose rather than using routinely collected data as in our study. Studies using routinely collected data are susceptible to missing data, although in our study over 99% of patients had smoking status recorded, 87% had ethnic group recorded, and over 82% had complete data for all five clinical values (table 2). We also used multiple imputation to deal with missing data. Other problems with routine data include coding errors and variable timing between measurements of risk factors because of differences in when patients present to their general practitioner. Advantages of using routinely collected data rather than a purposeful cohort include size, efficiency, better generalisability, and less susceptibility to selection bias or attrition bias.

We fitted several different models and carried out sensitivity analyses that showed some heterogeneity of results with variations in point estimates. The results are therefore sensitive to the assumptions made in the study design and modelling and have uncertainty; however, our findings were generally consistent across the different analyses for glitazones and gliptins.

### Conclusions

We have found clinically important differences in risk of cardiovascular disease, heart failure, and all cause mortality between different diabetes drugs alone and in combination compared with no drug treatment. Overall, use of gliptins or glitazones was associated with a decreased risk of heart failure, cardiovascular disease, and all cause mortality compared with non-use of these drugs. These results, which do not account for levels of adherence or dosage information and which are subject to confounding by indication, may have implications for prescribing of diabetes drugs.

**What is already known on this topic**

Cardiovascular disease and heart failure are major causes of morbidity and mortality in people with type 2 diabetes
Several diabetes drugs have been associated with an unexpected increased risk of heart failure during clinical trials and post-marketing surveillance raising concerns about overall risks and benefits
There is a need to quantify risks of clinical outcomes in large representative populations of people with type 2 diabetes prescribed these drugs over longer periods

**What this study adds**

Clinically important differences in risk of cardiovascular disease, heart failure, and all cause mortality were found between different diabetes drugs alone and in combination
Compared with non-use of gliptins, gliptin use was significantly associated with an 18% decreased risk of all cause mortality, a 14% decreased risk of heart failure, and no significant change in risk of cardiovascular disease
Compared with non-use of glitazones, glitazones were significantly associated with a 23% decreased risk of all cause mortality, a 26% decreased risk of heart failure, and a 25% decreased risk of cardiovascular disease

## Web extra

Extra material supplied by author

## Notes

We acknowledge the contribution of the practices that contribute to QResearch and Egton Medical Information Systems (EMIS), and the University of Nottingham for expertise in establishing, developing, and supporting the database. The data from hospital episode statistics used in this analysis are re-used by permission from the Health and Social Care Information Centre who retain the copyright. We thank the Office for National Statistics for providing the mortality data. ONS bear no responsibility for the analysis or interpretation of the data.

Ethical approval: This project was reviewed in accordance with the QResearch agreement with Trent MultiCentre Ethics Committee (reference 03/4/021).

Data sharing: The patient level data from the QResearch are specifically licensed according to its governance framework. See www.qresearch.org for further details.

Transparency: The lead author (JH-C) affirms that the manuscript is an honest, accurate, and transparent account of the study being reported, that no important aspects of the study have been omitted, and that any discrepancies from the study as planned have been explained.
