# Supplementary material for: Diabetes treatments and risk of heart failure, cardiovascular disease, and all cause mortality: cohort study in primary care
Source: BMJ. 2016 Jul 13;354:i3477. doi: 10.1136/bmj.i3477 (PMC4948032; doi:10.1136/bmj.i3477)
Supplement: Supplementary file 1 — Supplementary file: additional information [file hipj030247.ww1_default.pdf]

Supplementary table 1 Mean values prior to starting different medication combinations or at study entry for prevalent users

	age	Townsend score	HBA1C mmol/mol	BMI kg/m^2	Cholesterol/HDL ratio	SBP mmHg	Creatinine mol/l
No treatment	64.6	0.4	57.6	30.5	3.9	132.7	87.9
**Monotherapy**							
Metformin alone	64.1	0.5	60.0	30.8	3.8	132.3	83.2
Sulphonylureas alone	70.5	0.4	63.1	28.8	3.8	132.2	110.8
Insulin alone	66.4	0.3	66.6	29.6	3.9	130.2	118.5
Glitazones alone	66.8	-0.2	59.3	32.0	3.8	133.3	101.4
Gliptins alone	67.8	0.1	63.5	31.2	3.9	131.5	98.3
Other diabetes treatments alone	63.7	0.7	63.2	32.3	4.0	132.3	100.6
**Dual therapy**							
Metformin and sulphonylureas	65.5	0.7	65.3	30.3	3.8	132.9	87.7
Metformin and insulin	63.5	0.6	69.4	31.1	3.9	131.7	87.4
Metformin and glitazones	61.2	0.1	63.0	32.6	3.8	132.8	82.5
Metformin and gliptins	61.8	0.3	65.2	32.2	3.8	131.7	80.4
Metformin and other hypo	59.3	0.7	66.1	34.1	3.9	131.6	79.6
Sulphonylureas and insulin	70.7	0.3	74.4	29.2	4.0	131.2	134.2
Sulphonylureas and glitazones	70.2	0.1	65.9	31.0	3.8	133.4	112.8
Sulphonylureas and gliptins	70.0	0.2	70.2	30.7	3.9	132.5	105.9
Sulphonylureas and other hypo	66.0	0.4	70.6	32.8	4.1	131.4	106.7
**Triple therapy**							
Metformin, sulphonylureas and insulin	65.0	0.6	76.2	30.3	3.9	132.2	90.8
Metformin, sulphonylureas and glitazones	63.3	0.5	68.2	31.3	3.8	133.3	86.5
Metformin, sulphonylureas and gliptins	63.5	0.7	70.4	31.3	3.8	132.4	83.6
Metformin, sulphonylureas and other hypo	60.4	0.8	72.7	33.8	4.0	132.5	81.8
All other drug combinations	61.6	0.5	73.2	32.5	3.9	132.5	86.5

Supplementary Table 2a. Adjusted hazard ratios (95% CI) for each outcome (models A, B,C, D and E)

		Adjusted HR model A ±	*Adjusted HR model B* ±	*Adjusted HR model C* †	*Adjusted HR model D* †	*Adjusted HR model E*±
**All-cause mortality**	Glitazones	0.57 (0.53 to 0.62)	0.73 (0.67 to 0.79)	0.77 (0.71 to 0.84)	0.68 (0.61 to 0.77)	0.76 (0.67 to 0.86)
	Gliptins	0.73 (0.69 to 0.78)	0.81 (0.76 to 0.86)	0.82 (0.77 to 0.88)	0.71 (0.65 to 0.78)	0.80 (0.73 to 0.87)
	Metformin	0.43 (0.42 to 0.44)	0.57 (0.56 to 0.58)	0.59 (0.58 to 0.60)	0.48 (0.47 to 0.50)	0.62 (0.61 to 0.64)
	Sulphonylureas	0.95 (0.93 to 0.97)	1.09 (1.07 to 1.12)	1.10 (1.07 to 1.12)	1.31 (1.27 to 1.36)	1.21 (1.17 to 1.25)
	Insulin	1.45 (1.39 to 1.51)	1.44 (1.38 to 1.50)	1.47 (1.41 to 1.53)	1.57 (1.49 to 1.66)	2.16 (2.04 to 2.30)
	other hypo	0.67 (0.61 to 0.75)	0.77 (0.69 to 0.86)	0.82 (0.73 to 0.91)	0.82 (0.73 to 0.91)	0.80 (0.69 to 0.93)
	Gliptins*age				1.016 (1.010 to 1.022)	
	Gliptins*HBA1C				1.008 (1.004 to 1.011)	
	Glitazones*age				1.016 (1.007 to 1.024)	
	Glitazones*HBA1C				1.007 (1.003 to 1.012)	
	Insulin*age				0.991 (0.987 to 0.994)	
	Insulin*HBA1C				1.010 (1.008 to 1.012)	
	Metformin*age				1.018 (1.016 to 1.020)	
	Metformin*BMI				1.017 (1.013 to 1.020)	
	Metformin*HBA1C				0.996 (0.995 to 0.997)	
	Sulphonylureas*age				0.987 (0.985 to 0.989)	
**Heart failure**	Glitazones	0.80 (0.71 to 0.90)	0.79 (0.71 to 0.89)	0.74 (0.66 to 0.83)	0.74 (0.66 to 0.83)	0.66 (0.55 to 0.79)
	Gliptins	0.92 (0.83 to 1.01)	0.85 (0.77 to 0.93)	0.86 (0.78 to 0.95)	0.86 (0.78 to 0.95)	0.86 (0.75 to 0.98)
	Metformin	0.76 (0.73 to 0.79)	0.69 (0.67 to 0.72)	0.70 (0.68 to 0.73)	0.63 (0.59 to 0.66)	0.70 (0.67 to 0.73)
	Sulphonylureas	1.20 (1.16 to 1.25)	1.00 (0.96 to 1.04)	1.04 (1.00 to 1.08)	1.14 (1.07 to 1.20)	1.08 (1.01 to 1.15)
	Insulin	1.68 (1.55 to 1.82)	1.31 (1.21 to 1.42)	1.32 (1.22 to 1.43)	1.33 (1.22 to 1.44)	1.56 (1.37 to 1.78)
	Other hypo	1.09 (0.94 to 1.26)	0.94 (0.81 to 1.08)	0.92 (0.79 to 1.06)	0.91 (0.79 to 1.05)	0.91 (0.74 to 1.12)
	Metformin*age				1.010 (1.007 to 1.014)	

	Metformin*BMI				1.013 (1.007 to 1.019)	
	Sulphonylureas*age				0.993 (0.989 to 0.996)	
**cardiovascular disease**	Glitazones	0.81 (0.75 to 0.87)	0.72 (0.66 to 0.77)	0.75 (0.69 to 0.81)	0.75 (0.69 to 0.81)	0.69 (0.61 to 0.77)
	Gliptins	0.99 (0.93 to 1.06)	0.91 (0.85 to 0.98)	0.94 (0.88 to 1.00)	0.92 (0.86 to 0.99)	0.90 (0.82 to 0.98)
	Metformin	0.86 (0.84 to 0.89)	0.73 (0.71 to 0.75)	0.76 (0.74 to 0.78)	0.75 (0.72 to 0.77)	0.76 (0.74 to 0.79)
	Sulphonylureas	1.15 (1.12 to 1.19)	1.00 (0.97 to 1.03)	1.00 (0.97 to 1.03)	1.00 (0.98 to 1.04)	1.05 (1.01 to 1.10)
	Insulin	1.39 (1.31 to 1.48)	1.21 (1.13 to 1.29)	1.23 (1.15 to 1.31)	1.09 (1.02 to 1.18)	1.23 (1.11 to 1.37)
	Other hypo	1.06 (0.96 to 1.17)	0.91 (0.82 to 1.00)	0.95 (0.86 to 1.05)	0.94 (0.85 to 1.04)	0.99 (0.86 to 1.13)
	Gliptins*HBA1C				1.008 (1.004 to 1.011)	
	Glitazones*HBA1C				1.007 (1.003 to 1.012)	
	Insulin*HBA1C				1.018 (1.015 to 1.021)	
	Metformin*HBA1C				0.997 (0.996 to 0.999)	

Model A: adjusted for age, sex, ethnicity, deprivation, calendar year, duration of diabetes plus other diabetes drugs
Model B: model A + comorbidities (hypertension; cardiovascular disease; atrial fibrillation; chronic renal disease; rheumatoid arthritis; valvular heart disease; peripheral vascular disease) + existing complications (history of hypoglycaemia; hyperglycaemia; amputation; severe kidney disease; blindness) + use of other drugs (statins, aspirin, anticoagulants, diuretics, ACE inhibitors/angiotensin blockers, beta blockers, calcium channel blockers)
Model C (main paper): model B + clinical values (body mass index, cholesterol/HDL ratio, systolic blood pressure, serum creatinine, glycosylated haemoglobin)
Model D: model C + interaction terms shown in the table
Model E model C having excluded prevalent users of sulphonylureas (sample size for analysis 979,049)

Supplementary table 2b Hazard ratios for interaction terms at different values of age, HbA1c and BMI

All-cause mortality						
	Age (years)					
	**35**	**45**	**55**	**65**	**75**	**85**
Glitazones	0.43	0.51	0.59	0.69	0.81	0.95
Gliptins	0.45	0.53	0.62	0.72	0.85	1.00
Metformin	0.29	0.34	0.41	0.49	0.59	0.70
Sulphonylureas	1.91	1.67	1.47	1.29	1.13	0.99
Insulin	2.04	1.86	1.70	1.55	1.42	1.30
	**HbA1c**					
	**50**	**55**	**60**	**65**	**70**	**75**
Glitazones	0.63	0.65	0.68	0.70	0.72	0.75
Gliptins	0.65	0.68	0.70	0.73	0.76	0.79
Metformin	0.50	0.49	0.48	0.47	0.46	0.45
Insulin	1.41	1.48	1.56	1.64	1.72	1.81
	**BMI (kg/m^2)**					
	**23**	**25**	**30**	**35**	**40**	**45**
Metformin	0.42	0.44	0.47	0.52	0.56	0.61
**Heart failure**						
	Age (years)					
	35	45	55	65	75	85
Metformin	0.47	0.52	0.58	0.64	0.70	0.78
Sulphonylureas	1.39	1.30	1.21	1.13	1.05	0.98
	**BMI (kg/m^2)**					
	**23**	**25**	**30**	**35**	**40**	**45**
Metformin	0.57	0.59	0.62	0.67	0.71	0.76
**Cardiovascular disease**						
	**HbA1c**					
	**50**	**55**	**60**	**65**	**70**	**75**
Glitazones	0.70	0.72	0.75	0.77	0.80	0.83
Gliptins	0.84	0.88	0.91	0.95	0.99	1.03
Metformin	0.77	0.76	0.75	0.74	0.73	0.72
Insulin	0.90	0.98	1.07	1.17	1.28	1.40

Supplementary table 3 Adjusted hazard ratios with 95% confidence intervals for each outcome having excluded prevalent users of sulphonylureas (model G). Treatment categories are mutually exclusive.

	Adjusted HR heart failure†	*Adjusted HR Cardiovascular disease †*	*Adjusted HR all-cause mortality†*
No treatment	1.00	1.00	1.00
**monotherapy**			
Metformin alone±	0.70 (0.67 to 0.73)	0.77 (0.75 to 0.80)	0.65 (0.64 to 0.67)
Sulphonylureas alone±	1.16 (1.04 to 1.29)	1.12 (1.02 to 1.23)	1.50 (1.43 to 1.58)
Insulin alone±	1.45 (1.18 to 1.78)	1.07 (0.90 to 1.27)	2.27 (2.10 to 2.45)
Glitazones alone±	0.34 (0.14 to 0.81)	0.66 (0.42 to 1.05)	0.77 (0.54 to 1.09)
Gliptins alone±	0.79 (0.49 to 1.28)	1.17 (0.84 to 1.63)	0.99 (0.78 to 1.26)
Other drugs alone±	0.88 (0.50 to 1.55)	1.00 (0.66 to 1.51)	1.02 (0.78 to 1.34)
**Dual therapy**			
Metformin & sulphonylureas±	0.75 (0.69 to 0.82)	0.78 (0.74 to 0.83)	0.65 (0.62 to 0.69)
Metformin & insulin±	1.25 (0.99 to 1.58)	0.89 (0.73 to 1.09)	1.18 (1.02 to 1.37)
Metformin and glitazones±	0.49 (0.38 to 0.63)	0.47 (0.40 to 0.55)	0.58 (0.49 to 0.68)
Metformin and gliptins±	0.68 (0.56 to 0.83)	0.69 (0.61 to 0.79)	0.54 (0.47 to 0.62)
Metformin and other hypo±	0.80 (0.61 to 1.05)	0.73 (0.60 to 0.90)	0.54 (0.43 to 0.68)
Sulphonylureas and insulin±	1.02 (0.59 to 1.76)	1.73 (1.21 to 2.47)	2.71 (2.25 to 3.27)
Sulphonylureas and glitazones±	0.83 (0.46 to 1.50)	0.82 (0.51 to 1.32)	0.80 (0.53 to 1.21)
Sulphonylureas and gliptins±	0.68 (0.40 to 1.14)	0.79 (0.53 to 1.18)	0.85 (0.65 to 1.11)
Sulphonylureas and other hypo±	0.34 (0.05 to 2.38)	1.66 (0.83 to 3.33)	1.41 (0.76 to 2.62)
**Triple therapy**			
Metformin, sulphonylureas and insulin±	1.42 (1.00 to 2.01)	1.13 (0.84 to 1.50)	1.87 (1.52 to 2.30)
Metformin, sulphonylureas and glitazones±	0.54 (0.37 to 0.79)	0.66 (0.53 to 0.82)	0.46 (0.34 to 0.62)
Metformin, sulphonylureas and gliptins±	0.60 (0.46 to 0.79)	0.65 (0.55 to 0.77)	0.54 (0.45 to 0.65)
Metformin, sulphonylureas and other hypo±	0.47 (0.26 to 0.85)	0.63 (0.43 to 0.90)	0.39 (0.23 to 0.64)
All other drug combinations±	0.63 (0.44 to 0.88)	0.85 (0.69 to 1.04)	0.73 (0.58 to 0.92)

± compared with periods not on any treatment.

†Hazard ratios adjusted for the following: sex; age; calendar year, duration since diagnosis of diabetes (5 levels); ethnicity (9 levels) Townsend deprivation score smoking status (5 levels) use of anticoagulant thiazide ace angiotensin 2 blockers; calcium channel blockers; statins; aspirin; existing complications (blindness, hyperglycaemic coma; hypoglycaemia; amputation; severe kidney failure); hypertension; cardiovascular disease; atrial fibrillation; chronic renal disease; rheumatoid arthritis; valvular heart disease; peripheral vascular disease; body mass index; systolic blood pressure; HBA1C; serum creatinine; cholesterol/HDL ratio.
